# High-quality and Fast Mapping of a Focal Atrial Tachycardia Arising from Koch’s Triangle

**DOI:** 10.19102/icrm.2021.120114S

**Published:** 2021-01-15

**Authors:** Paolo China, Levio Quinto, Raffaele Vitale, Andrea Corrado, Elena Marras, Sakis Themistoclakis

**Affiliations:** ^1^Unit of Electrophysiology and Cardiac Pacing, Department of Cardiothoracic & Vascular Medicine, Ospedale dell’Angelo-ULSS3 Serenissima, Mestre-Venice, Italy

**Keywords:** Dynamic mapping, focal atrial tachycardia, high-density mapping

Focal atrial tachycardias arise with different mechanisms, including from atrial structures where ablation is dangerous to perform and detailed mapping is necessary but time-consuming. Recently, a new software, EnSite™ LiveView Dynamic Display, was introduced to provide a fast, real-time beat-to-beat analysis of electrical information.

A 43-year-old woman without previous illness presented in our department with a long history of palpitations and supraventricular tachycardia interrupted with adenosine. We performed an electrophysiological study in our electrophysiology laboratory, confirming with pacing maneuvers the diagnosis of atrial tachycardia conducted with right bundle branch block aberrancy and initiated mapping using the Ensite Precision™ mapping system and the Advisor™ HD Grid Mapping Catheter, Sensor Enabled™. At the beginning, we adopted the traditional static local activation map (15 minutes) to identify a macro-region of interest in the septum near the triangle of Koch **(Video 1, part 1)**. Afterward, the EnSite™ Liveview software was used to more quickly (80 seconds) and precisely localize the site of earlier local atrial activation at the coronary sinus ostium **(Figure 1 and Video 1, part 2)**. Using a Blazer™ 4-mm ablation catheter (Boston Scientific, Natick, MA, USA) in this single site, we interrupted the atrial tachycardia after four seconds of radiofrequency energy (temperature control 50 W, 60°) **(Video 1, part 3)** with a few irritative junctional beats; two additional lesions were delivered to consolidate lesion formation at the site of successful termination (average: 39 W, 56°C).

Two conclusions can be drawn from this case. First, detailed mapping using the Advisor™ HD Grid catheter yielded a precise map that permitted us to create less lesions, avoiding further dangerous and unnecessary ones. Second, this case demonstrated utility of the new EnSite™ Liveview software in terms of its capacity for rapid mapping.

## Figures and Tables

**Figure 1:**
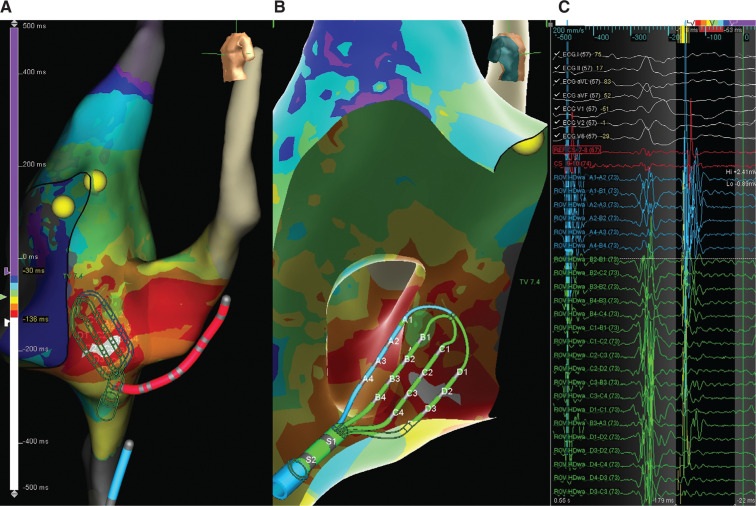
Traditional electroanatomical activation map (**A:** left anterior oblique view; **B:** right anterior oblique view). **C:** Signals recorded with the Advisor™ HD Grid during the focal atrial tachycardia in the Koch’s triangle region.

**Video 1:** Part 1: Traditional static local activation time mapping with the Advisor™ HD grid catheter. Part 2: LiveView dynamic mapping (left panel) as compared with previous static local activation time mapping (right panel). Part 3: First radiofrequency energy delivery interrupted atrial tachycardia after four seconds.

